# Antigen avoidance and environmental inhalation challenge for successful diagnosis of fibrotic hypersensitivity pneumonitis mimicking idiopathic pulmonary fibrosis

**DOI:** 10.1016/j.rmcr.2022.101737

**Published:** 2022-09-13

**Authors:** Yasuhiro Ito, Seiichi Miwa, Hiroshi Hayakawa, Tomoko Oshima, Tatsuru Eihuku, Eriko Iwaizumi, Hisano Ohba, Kaoru Fujita, Miho Kanai, Masahiro Shirai

**Affiliations:** Department of Respiratory Medicine, National Hospital Organization, Tenryu Hospital, Hamamatsu, Japan

**Keywords:** Fibrotic hypersensitivity pneumonitis, Farmer's lung, Idiopathic pulmonary fibrosis, Antigen avoidance, Inhalation challenge

## Abstract

A 77-year-old man was initially diagnosed with idiopathic pulmonary fibrosis (IPF) and treated with anti-fibrotic nintedanib. Despite undergoing anti-fibrotic treatment for one year, his condition remained unstable. The patient was admitted to our hospital for exertional dyspnea. We performed an exposure assessment, including 2-week antigen avoidance and an environmental inhalation challenge, and successfully re-diagnosed him with fibrotic hypersensitivity pneumonitis (HP), known as chronic farmer's lung. Adding oral glucocorticoids to the nintedanib treatment improved his condition. Although antigen avoidance and environmental inhalation challenge tests are not standardized, they may be useful for diagnosing fibrotic HP when properly applied.

## Highlights

• Clinical features of fibrotic HP and IPF are often indistinguishable.
• Antigen avoidance is crucial in the diagnosis and management of fibrotic HP.
• Environmental inhalation challenge is useful when applied properly.
• The seasonal change of clinical parameters helps suspecting fibrotic HP.

## 1 Introduction

Fibrotic hypersensitivity pneumonitis (HP), also known as chronic HP, is an immunologically mediated fibrotic interstitial lung disease (ILD) that develops in susceptible individuals after inhalation of the inciting antigens [1,2]. Unlike nonfibrotic HP, fibrotic HP has a poor prognosis and is often misdiagnosed as idiopathic pulmonary fibrosis (IPF), due to similarities in the clinical presentation [3]. Given the pathogenesis of fibrotic HP, antigen avoidance and inhalation challenge are considered fundamentally important tests for its diagnosis. However, these tests are not standardized, and a few case reports demonstrating their usefulness have been published.

Here, we report a case successfully diagnosed with fibrotic HP/chronic farmer's lung mimicking IPF by exposure re-assessment including a detailed interview, antigen avoidance, and an environmental inhalation challenge. Depending on the individual situation, antigen avoidance and environmental inhalation challenges can be useful tests for the diagnosis of fibrotic HP.

## 2 Case presentation

**Fig. 1:**
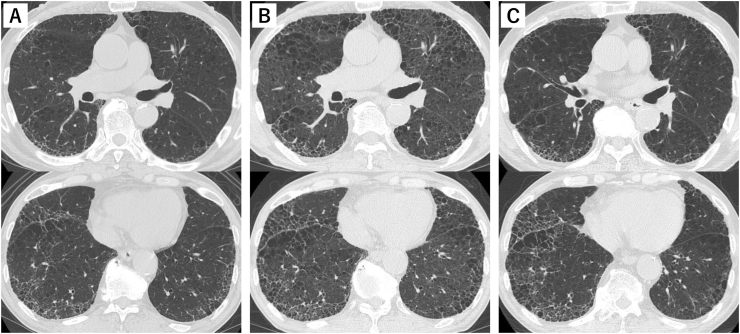
High resolution computed tomography demonstrated emphysema, reticulation, and honeycombing without nodular shadows and mosaic pattern suggesting fibrotic hypersensitivity pneumonitis. (A) Before admission. (B) At admission, the density of lung field increased slightly and diffusely. (C) Two years after addition of oral glucocorticoid, the density of lung field improved.

**Fig. 2:**
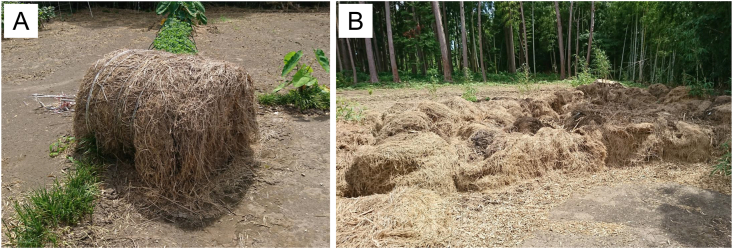
Environmental assessment. (A) A roll of hay in the patient's farm. (B) The compost area.

A 77-year-old man with interstitial pneumonia was admitted to our hospital after 2 weeks of exertional dyspnea following hay spreading on his taro field for 3 days. Five years prior to admission, he had been diagnosed with interstitial pneumonia. At that time, his bronchoalveolar lavage fluid (BALF) contained 11% lymphocytes with a CD4/CD8 ratio of 1.65, and a transbronchial lung biopsy revealed interstitial pneumonia with fibroblastic foci. The interstitial pneumonia progressed gradually with slight worsening in winters and improving. One year before admission to our hospital, the interstitial pneumonia slightly worsened and nintedanib was administered to treat clinical IPF. The IPF had been diagnosed based on the presence of the interstitial pneumonia (UIP) pattern on high-resolution computed tomography (HRCT) (Fig. 1A), and the absence of collagen vascular disease. However, the administered treatment did not stabilize the disease. The patient's medical history included hypertension, spiral canal stenosis, and benign prostatic hyperplasia. He was a smoker from the age of 20–72 years, and worked as a sand caster until the age of 72 years. As a hobby, he grew vegetables. Eight years before admission, he started to make compost from hay in an area approximately 100 m away from his house (Fig. 2). Over the past five years, the farming had become more extensive than we thought. He wore a dust mask while making compost.

On admission, his body temperature was 36.5 °C, and oxygen saturation (SpO_2_) was 92% when breathing ambient air. Lung auscultation revealed fine crackles and he also had clubbed fingers. Chest radiography revealed bilateral reticular shadows predominating in the lower lung fields, with slight enhancement of ground-glass opacity. Chest HRCT showed neither nodular shadows nor a mosaic pattern suggestive of fibrotic HP. The density of both lung fields appeared diffusely increased when compared with that of 2 years previous (Fig. 1B). The patient's white blood cell count (WBC) was 4.76 × 10^9^/L, lactate dehydrogenase (LDH) level was 291 IU/L, and C-reactive protein (CRP) level was 1.92 mg/dL. The levels of ILD markers Krebs von den Lungen-6 (KL-6) and pulmonary surfactant protein-D (SP-D) were 847 U/mL (reference range, up to 500 U/mL) and 330 ng/mL (reference range, up to 110), respectively. Pulmonary function tests revealed a decreased diffusing capacity of the lung for carbon monoxide (DLCO) (40.8% of predicted value), with a normal vital capacity and a normal ratio of forced expiratory volume in 1 s (FEV_1_) to forced vital capacity (FVC). The partial pressure of oxygen (PaO_2_) in ambient air was 62.8 Torr.

**Fig. 3:**
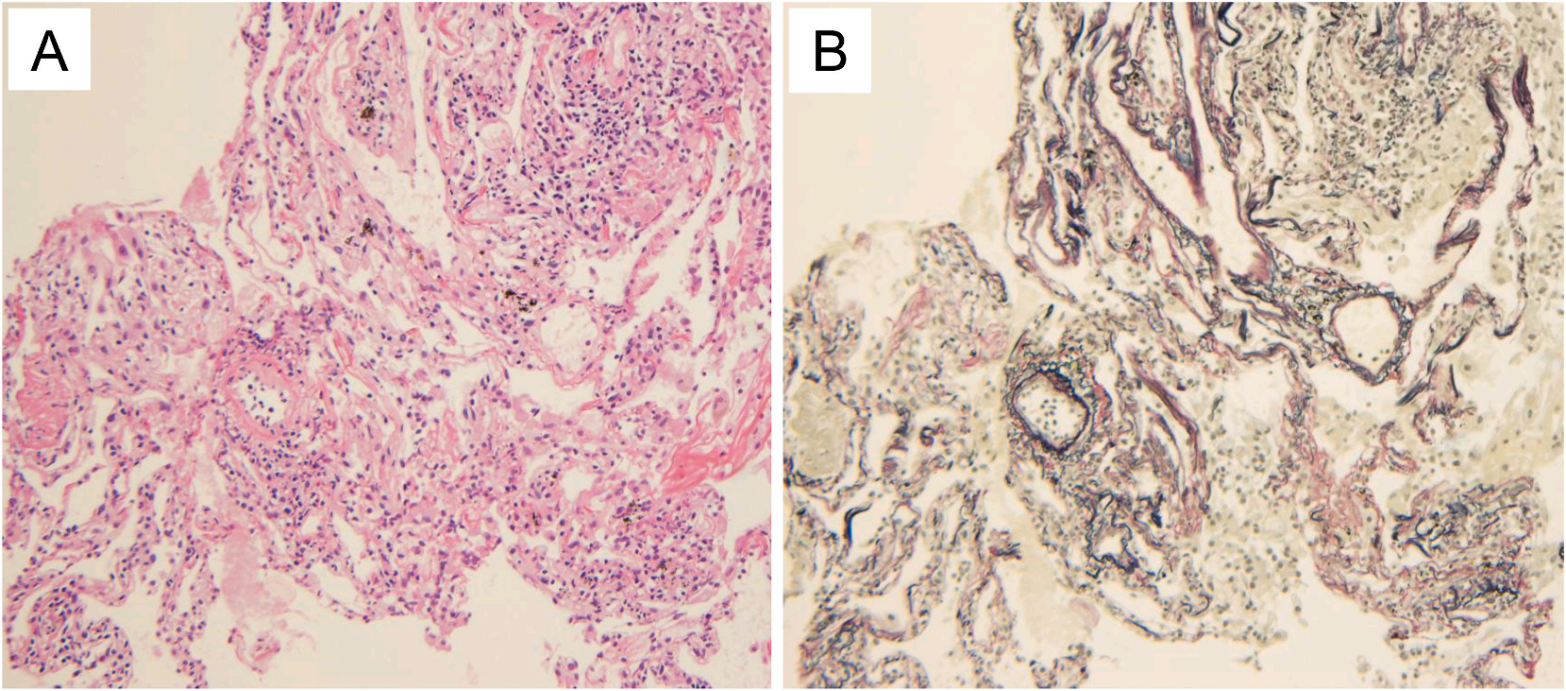
Transbronchial lung biopsy shows mild alveolitis and fibrosis without granuloma. (A) Hematoxylin and Eosin staining, x200. (B) Elastica van Gieson staining, x200.

**Fig. 4:**
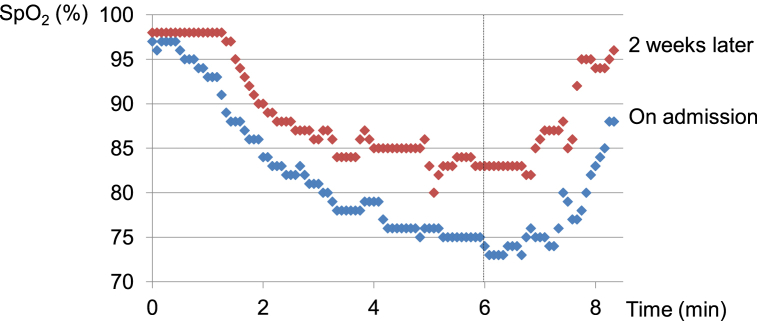
Time course of SpO_2_ during the 6-min walk test. Walking distance was 375 m (both on admission and 2 weeks later). SpO_2_: oxygen saturation.

A bronchoscopy was performed on the day after admission, and BALF (right B5) lymphocytosis (27.8%) was found with a CD4/CD8 ratio of 34.25. A transbronchial lung biopsy (right B3, B4, and B8) revealed alveolitis without granulomas (Fig. 3). Because of BALF lymphocytosis and fluctuation of symptoms, we suspected fibrotic HP due to hay and observed the patient without therapy at our hospital as an antigen avoidance test. After 2 weeks, LDH, CRP, PaO_2_, alveolar-arterial oxygen difference (A-aDO2), and oxygen desaturation on the 6-min walk test had improved (Table 1, Fig. 4). Subsequently, an environmental inhalation challenge test was conducted. The patient returned to his farm and moved a hay roll into the composting area for 30 min. His WBC and A-aDO2 levels were elevated 10 h after provocation, and the CRP level was elevated 24 h after provocation (Table 2). Samples of hay from the farm were analyzed and revealed an abundance of *Aspergillus* species (*Aspergillus fumigatus*, *Aspergillus niger*, and *Aspergillus terreus*).

**Table 1:** Antigen avoidance test.

	Admission	Antigen Avoidance (2 weeks)
Body Temperature (°C)	36.5	36.6
WBC (/μl)	4760	5280
CRP (mg/dl)	1.92	0.30
LDH (IU/L)	291	192
KL-6 (U/ml)	847	1230
SP-D (ng/ml)	330	297
PaO_2_ (torr)	62.8	71.1
A-aDO_2_ (torr)	41.8	21.9
VC (L)	3.18	3.24
symptom	exertional dyspnea, cough	no symptom

WBC: White blood cell count, CRP: C-reactive protein, LDH: lactate dehydrogenase, KL-6: Krebs von den Lungen-6, SP-D: pulmonary surfactant protein-D, PaO_2_: partial pressure of oxygen, A-aDO_2_: alveolar-arterial oxygen difference, VC: vital capacity.

**Table 2:** Environmental inhalation challenge test.

	Before provocation	10h after provocation	24h after provocation	Δ
Body Temperature (°C)	36.4	36.8	36.6	0.4
WBC (/μL)	5280	8020	6000	2740
CRP (mg/dL)	0.30	0.39	0.86	0.56
PaO_2_ (torr)	71.1	71.1	72.2	1.1
A-aDO2	21.9	29.4	22.5	7.5
VC (L)	3.24	3.25	3.33	0.09
symptom	no symptom	no symptom	no symptom	

WBC: white blood cell count, CRP: C-reactive protein, PaO_2:_ Partial pressure of oxygen, A-aDO_2_: alveolar-arterial oxygen difference, VC: vital capacity, Δ: difference.

Based on the clinical course, chest imaging, lymphocytosis in BALF, improvement with antigen avoidance, and worsening with the environmental inhalation challenge test, we diagnosed the patient with fibrotic HP caused by exposure to hay, often termed as chronic farmer's lung. Precipitated antibodies against *Aspergillus* species, as well as *Saccharopolyspora rectivirgula* and *Thermoactinomyces vulgaris*, which are common causative antigens of farmer's lung, were negative.

**Fig. 5:**
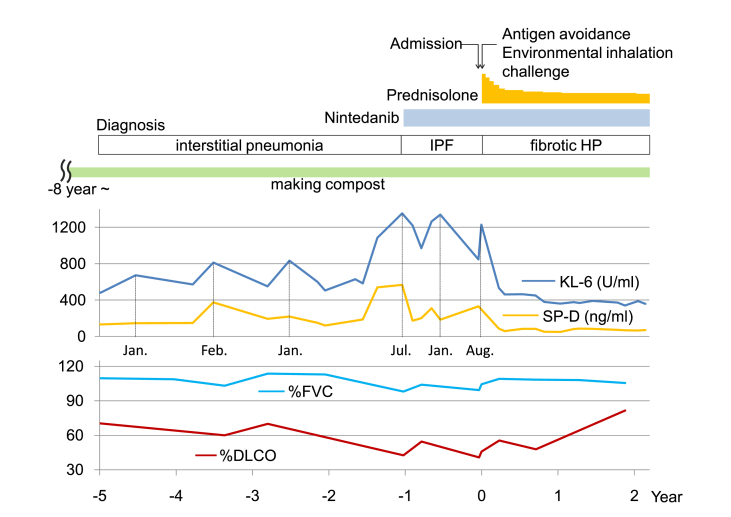
Clinical course. Addition of prednisolone to Nintedanib decreases serum KL-6 and SP-D levels, and increases %DLCO. IPF: idiopathic pulmonary fibrosis, HP: hypersensitivity pneumonitis, KL-6: Krebs von den Lungen-6, SP-D: pulmonary surfactant protein-D, DLCO: diffusing capacity of the lung for carbon monoxide, FVC: forced vital capacity.

The patient's condition worsened over the years, but he refused to avoid the farm environment. Hence, we instructed the patient to wear dust masks not only when making compost but also when handling hay. We added 20 mg oral prednisolone daily to his treatment regimen. Subsequently, the dose of oral prednisolone was gradually reduced to 6.5 mg daily, and the patient's condition remained stable for over 2 years (Figs. 1C and 5).

## 3 Discussion

In this report, we present the case of a patient who was initially diagnosed with IPF and later re-diagnosed with chronic farmer's lung by exposure re-assessment. Farmer's lung is one of the most common forms of HP, and is caused by inhalation of microorganisms from moldy hay or straw. While this disease typically presents in farmers, people other than farmers who are exposed to the farm environment can also be affected. It is important to obtain information regarding not only residential and occupational environments but also avocational settings from patients suspected of having fibrotic HP.

The most common antigen leading to farmer's lung is a thermophilic actinomycete*, Saccharopolyspora rectivirgula*, which contaminates farms. In addition, farmers are also exposed to other thermophilic actinomycetes, like *Thermoactinomyces vulgaris*, and fungi, like *Aspergillus* species, that can also cause farmer's lung [4]. There are some case reports on farmer's lung caused by *Aspergillus* species found in compost [5,6]. In our case, although precipitated antibodies to *Aspergillus* species were negative, we found an abundance of *Aspergillus* species in hay and suspected that *Aspergillus* species was the causative antigen.

Diagnosis of fibrotic HP is often difficult because of its similarity to IPF, often resulting in misdiagnosis. Morell et al. reported that 43% of patients diagnosed with IPF based on the 2011 guidelines had a subsequent diagnosis of chronic HP upon re-examination [3].

Although clinical improvement with antigen avoidance is helpful in the diagnosis of HP, the absence of clinical improvement does not exclude it [2,7]. Generally, antigen avoidance is less useful for the diagnosis of fibrotic HP than that of nonfibrotic HP. Nonfibrotic HP arises from short-term exposure to high levels of antigens, whereas fibrotic HP arises from long-term exposure to low levels of antigens, and continuously decreases FVC in some patients even after antigen avoidance [8]. Tsutsui et al. reported that a 2-week in-hospital antigen avoidance test for chronic HP had a sensitivity and specificity of 51.0% and 80.7%, respectively, and concluded that the low sensitivity might be due to the relatively short duration of antigen avoidance [9]. In our case, improvement of vital capacity (VC), WBC, and KL-6 did not meet the positive criteria reported by Tsutsui et al., but the other clinical parameters clearly improved. Therefore, we considered the antigen avoidance test to be positive.

Clinical worsening with inhalation challenge under controlled conditions also has a high diagnostic value for HP. Inhalation challenges include environmental inhalation challenges and specific inhalational challenges (SIC). The environmental inhalation challenge is a re-exposure of the patient to the environment and can be easily performed in clinical practice. The SIC is a more direct exposure of the patient to precisely identified and purified inciting antigens in laboratory settings. Because there are no standardized procedures for environmental inhalation challenges and SIC, recent guidelines for the diagnosis of HP do not recommend inhalation challenges [7]. There are only a few reports on the usefulness of environmental inhalation challenge in fibrotic HP. According to a nationwide survey in Japan that included 222 patients with chronic HP, the positivity rate was 59.6% [10]. When it comes to adaptation of environmental inhalation challenge to fibrotic HP, we must take a risk of exacerbation into consideration. Therefore, environmental inhalation challenge may not be required for some patients with fibrotic HP who can avoid the causative antigen and, for example, is adapted to the following situation; a patient with suspected fibrotic HP (1) who cannot avoid the causative antigen completely, (2) who has some symptoms even after antigen avoidance, or (3) who wants to identify the causative antigen or environment. Regarding the procedure of environmental inhalation challenge for fibrotic HP, if short-term exposure does not induce symptoms, we recommend long-term exposure. It is reported that the temporal evolution of exposure-related symptoms may take days or weeks to recur [11]. In terms of SIC, two retrospective studies have reported the diagnostic utility of SIC in chronic bird-related HP. Ishizuka et al. showed that the SIC prediction score (ΔWBC [%] + 2 × ΔP [A-a] O2 [mmHg], with a cut-off value of 35) had high sensitivity (92.9%) and specificity (94.7%) [12]. Okuda et al. indicated that mean changes in CRP level (0.32 mg/dL) and A-aDO_2_ (7.8 Torr) were candidate parameters [13]. Although our case was neither chronic bird-related HP nor used by SIC, according to the criteria, the environmental inhalation challenge was interpreted as positive.

The serum-specific immunoglobulin G (SS-IgG) test is used to identify causative antigens. However, its diagnostic utility is controversial because a positive SS-IgG result merely shows that the patient is likely to be exposed to the antigen, and does not indicate the cause of HP [1,2]. The positivity rate of SS-IgG in chronic HP varies depending on antigen type, stage of disease, smoking, duration, and level of exposure (35.0–75.3%) [10,14]. In our case, SS-IgG, expected to be the inciting antigens, were negative.

Serum KL-6 is a useful biomarker for ILD, including HP. In addition, according to the report by Ohnishi et al. [15], seasonal variation of serum KL-6 levels was observed, particularly in patients with HP, which could indicate the possibility of HP during the clinical course of ILD. In our case, these seasonal changes helped us suspect fibrotic HP.

The management of fibrotic HP should begin with antigen avoidance, which is the mainstay treatment. Elimination of the causative antigen is crucial to slow the progression of the disease in patients with fibrotic HP. Although it can be difficult to achieve in practice, we must make continuous efforts to strongly recommend antigen avoidance and educate individuals on how to mitigate antigen exposure. The use of a respiratory protective device is a practical preventive measure [4]. Kusaka et al. reported that the use of a dust mask during farming work was effective in preventing the recurrence of farmer's lung [16]. If the antigen is unavoidable or lung inflammation persists despite removal of the antigen, anti-inflammatory treatment (glucocorticoids, immunosuppressive agents), followed by antifibrotic agents, should be considered [17].

Recently, the anti-fibrotic agent nintedanib was shown to significantly reduce the annual decline in FVC in patients with progressive fibrosing (PF)-ILD (non-IPF) compared to placebo [18]. Subgroup analysis of fibrotic HP also showed a similar tendency [19]. Our case did not exhibit a decline in FVC due to emphysema, but there was a decline in DLCO along with worsening respiratory symptoms, and increased extent of fibrosis. Therefore, we categorized the condition as PF-ILD and continued nintedanib.

## 4 Conclusion

The clinical features of fibrotic HP and IPF are often indistinguishable, but the management of fibrotic HP is fundamentally different from that of IPF. Exposure assessment, including antigen avoidance and subsequent inhalation challenge, is crucial for diagnosing fibrotic HP. However, because fibrotic HP occurs after long-term exposure to a wide variety of antigens, these tests are not standardized. Thus, further studies, including additional case series, are required for antigen avoidance and inhalation challenge tests to be useful in the diagnosis of fibrotic HP.

## Funding

None.

## Author contributions

YI, SM, HH, TO, TE, EI, HO, KF, MK, and MS contributed to decision of treatment, collecting clinical data, and data analysis. YI and SM wrote the manuscript. HH evaluated the pathological findings. All authors read and approved the final manuscript.

## Declaration of competing interests

The authors declare that they have no known competing financial interests or personal relationships that could have appeared to influence the work reported in this paper.
